# Cycling Stability of Lithium‐Ion Batteries Based on Fe–Ti‐Doped LiNi_0.5_Mn_1.5_O_4_ Cathodes, Graphite Anodes, and the Cathode‐Additive Li_3_PO_4_

**DOI:** 10.1002/advs.202301874

**Published:** 2023-06-22

**Authors:** Pirmin Stüble, Marcus Müller, Thomas Bergfeldt, Joachim R. Binder, Andreas Hofmann

**Affiliations:** ^1^ Institute for Applied Materials Karlsruhe Institute of Technology D‐76344 Eggenstein‐Leopoldshafen Germany; ^2^ Helmholtz Institute Ulm D‐89081 Ulm Germany

**Keywords:** additive, full cells, LiNi_0.5_Mn_1.5_O_4_, lithium phosphate, LNMO

## Abstract

This study addresses the improved cycling stability of Li‐ion batteries based on Fe–Ti‐doped LiNi_0.5_Mn_1.5_O_4_ (LNMO) high‐voltage cathode active material and graphite anodes. By using 1 wt% Li_3_PO_4_ as cathode additive, over 90% capacity retention for 1000 charge–discharge cycles and remaining capacities of 109 mAh g^−1^ are reached in a cell with an areal capacity of 2.3 mAh cm^−^^2^ (potential range: 3.5–4.9 V). Cells without the additive, in contrast, suffer from accelerated capacity loss and increase polarization, resulting in capacity retention of only 78% over 1000 cycles. An electrolyte consisting of ethylene carbonate, dimethyl carbonate, and LiPF_6_ is used without additional additives. The significantly improved cycling stability of the full cells is mainly due to two factors, namely, the low Mn^III^ content of the Fe–Ti‐doped LNMO active material and the use of the cathode‐additive Li_3_PO_4_. Crystalline Li_3_PO_4_ yields a drastic reduction of transition metal deposition on the graphite anode and prevents Li loss and the propagation of cell polarization. Li_3_PO_4_ is added to the cathode slurry that makes it a very simple and scalable process, first reported herein. The positive effects of crystalline Li_3_PO_4_ as electrode additive, however, should apply to other cell chemistries as well.

## Graphical abstract

In this study, it is shown that spinel‐based cathode materials can exhibit remarkable long‐term stability in full cells with graphite anodes. By using an Fe–Ti‐doped LiNi_0.5_Mn_1.5_O_4_ material with a low Mn(III) content and lithium phosphate powder as a cathode additive, over 90% capacity retention over 1000 cycles is achieved with a standard carbonate‐based electrolyte.

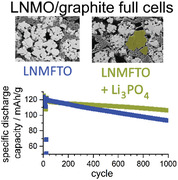

## 1 Introduction

**Figure 1:**
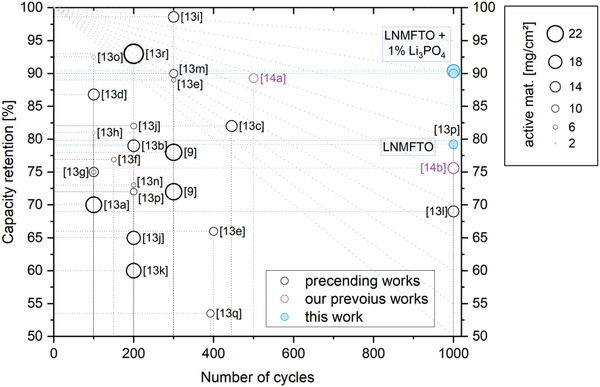
Graphical representation of the capacity retention in LNMO–graphite cells, with cathode active material loadings corresponding to the size of the marker. Black circles correspond to literature‐known data,^[^ ^9^, ^13^ ^]^ violet circles represent our previous works^[^ ^14^ ^]^ and the results of this paper are marked in light blue.

Spinel‐type lithium nickel manganese oxide materials have a three‐decade history in battery research. LiNi_0.4_Mn_1.6_O_4_ was first investigated in 1991^[^ ^1^ ^]^ and lithium manganese nickel oxide with the common composition LiNi_0.5_Mn_1.5_O_4_ (LNMO) was described in 1996.^[^ ^2^ ^]^ Despite its excellent energy density and cheaper raw materials, LNMO cathode active materials (CAMs) never were able to leap out of the shadow of the layered oxides like LiCoO_2_, which was discovered in 1980^[^ ^3^ ^]^ and commercially available in 1990.^[^ ^4^ ^]^ Nowadays, the structurally related lithium nickel manganese cobalt oxides discovered in 2001,^[^ ^5^ ^]^ together with lithium nickel cobalt aluminum oxides are dominating CAMs for high power applications.^[^ ^6^ ^]^ Despite many efforts, there is still a lot of contradictions and confusion about LNMO, for example concerning the Mn^III^ content, cation order, secondary phases and allegedly existing oxygen defects. Several aspects have only recently been addressed on the basis of systematic experimental work and an exact chemical equation for the formation of LNMO.^[^ ^7^ ^]^ The main reason for the lack of commercial success of LNMO materials, however, still is considered to be the strong degradation in LNMO/graphite cells. And indeed, the excellent results of LNMO‐Li cells, which run stably over more than 2000 cycles (see ref. [8], and references therein), could not even remotely be transferred to full cells. A detailed comparison of LNMO with other battery materials discussing, e.g., cost and sustainability and the reasons for the lack of commercialization of LNMO was given by the group of Ying Shirley Meng in 2020.^[^ ^9^ ^]^ The work also provides an overview of research papers on LNMO materials cycled against graphite. An enhanced graphical representation of preceding and current studies is shown in **Figure** **1**. When trying to figure out the most critical factors for the rapid degradation of LNMO versus graphite full cells, it becomes obvious that so far, no consensus has been reached regarding two central points, namely suitable electrolytes and the LNMO crystal chemistry.

The most controversial topic is probably electrolyte stability. Standard electrolytes based on organic carbonates are described to decompose rapidly as a consequence of a high cell voltage of >4.7 V versus Li/Li^+^, which are necessary due to on the energy levels of the Ni redox couples.^[^ ^10^ ^]^ In contrast, R. Jung et al. found that unlike nickel manganese cobalt oxide materials, LNMO itself hardly causes any electrolyte oxidation at potentials ≤5.0 V.^[^ ^11^ ^]^ Accordingly, electrolyte decomposition should not hinder stable cycling of LNMO–graphite cells at room temperature. In another publication of the Gasteiger group^[^ ^12^ ^]^ it is shown that traces of water substantially increase the anodic oxidation of carbon and electrolyte at cell voltages ≥4.6 V. Traces of water thus pose a significant challenge for the implementation of LNMO cathode material and could be the reason for the deviating findings on electrolyte stability.

A second controversy, which in our opinion received too little attention, relates to the crystal chemistry of LNMO. It is generally assumed that disordered LNMO has superior cycling properties. Unfortunately, the vast majority of disordered LNMO materials found in literature are quite Mn^III^ rich. Typical LNMO phase compositions of such disordered LNMO materials are ranging from LiNi_0.46_Mn_1.54_O_4_ to LiNi_0.43_Mn_1.57_O_4_ and thus contain 5%–9% Mn^III^.^[^ ^7^ ^]^ Mn^III^, however, is generally assumed to play a central role in full cell degradation. Mn^III^ ions disproportionate to Mn^IV^ and Mn^II^, and Mn^II^ readily dissolves in the electrolyte. Mn^II^ is then deposited on the graphite anode leading to Li‐consuming parasitic side reactions.^[^ ^15^ ^]^ In a previous work on Fe–Ti‐doped LNMO materials, however, no effect of cation order on the cycling stability of the doped LNMO material was found, suggesting that the effect of cation order might be overestimated. Simultaneously, however, superior full cell cycling stability for the LNMFTO material containing only 4% Mn^III^ was found.^[^ ^14a^ ^]^ It has also been shown that disordered LNMO materials with significantly reduced Mn^III^ content can be obtained and it was suggested that such materials should be best suited for the use in LNMO versus graphite full cells.^[^ ^7^ ^]^

Less controversial challenges with LNMO materials cycled against graphite electrodes are the following:

- Despite a theoretical specific capacity in the case of LNMO of 147 mAh g^−1^, the practical specific capacity is barely more than 120 mAh g^−1^ as a result of secondary phases of LNMO materials^[^ ^7^ ^]^ and Li consuming solid electrolyte interface (SEI) formation (see below).
- The loading of the electrolyte material is often limited due to the damage of LNMO electrodes during cycling, e.g., by crack formation and loss of electronic contact during cycling.^[^ ^9^, ^13^ ^]^

In recent years, these issues are addressed in the following manner:

- Specific additives are added to the electrolytes. This can be, for example, lithium bis(oxalato)borate or lithium difluoro(oxalato)borate, which improve the SEI or CEI layers, respectively, and can thus cause a reduction in metal deposition on the anode, for example. An improved SEI layer also improves the oxidation resistance of the electrolyte.^[^ ^14b^ ^]^ In principle, it is difficult to compare additives used in different LNMO materials with each other, because the effects of the additive may be different and also the aging of the cathode material differs. In this case, it is not trivial to clearly identify aging phenomena caused by the additive.
- The use of carbonate‐free solvents should also increase the oxidation resistance of the electrolyte. For example, instead of carbonates, sulfones are used, which enable a higher voltage limit.^[^ ^13l^ ^]^
- The consumption of Li during the buildup of the boundary layers (SEI and CEI) is partially attempted to be balanced with Li sources in the cell. For example, lithium oxalate can release Li^+^ ions above certain voltage limits.^[^ ^16^ ^]^ It is also being considered to use prelithiated graphite^[^ ^13f^ ^]^ or LNMO^[^ ^13r^ ^]^ to compensate for the Li loss.
- LNMO active particles are coated to ensure a reduced bleeding of metal ions and a better stabilization of the electrolyte.^[^ ^17^ ^]^ Such coatings can be done with inactive as well as active materials (e.g., Li_3_PO_4_^[^ ^18^ ^]^ or Zeolithes^[^ ^13q^ ^]^).
- The graphite anode can be coated with Al_2_O_3_ via atomic layer deposition which can reduce the Li consuming parasitic reactions significantly.^[^ ^13i^ ^]^ Although a considerable improvement in cycle stability can be achieved with this approach (see Figure 1), it must be mentioned that considerable effort is required to achieve such a layer. Also, it is no longer possible to refer to a graphite surface, since it is completely coated with a different material (e.g., Al_2_O_3_). Unfortunately, a scalability at reasonable costs is not achievable with this method.

Nevertheless, all these attempts to eliminate the issues of LNMO–graphite full cells also involve drawbacks. For example, the structure of the interfacial layers must be completely redesigned when other solvents are used. Prelithiation of graphite is also problematic, as it significantly increases the sensitivity to water and oxygen, so that process steps have to be adapted and modified at high cost. Coatings may result in significant overcharging due to the enhanced ohmic resistance.

Beside the systematic screening of additives,^[^ ^14b^ ^]^ our group recently presented a Ti–Fe doped LNMO material with a theoretical composition of LiNi_0.5_Mn_1.37_Fe_0.1_Ti_0.03_O_3.95_ (LNMFTO), which already showed significantly improved cycling stabilities compared to previous studies^[^ ^14a^ ^]^ (see violet circles in Figure 1). Based on these preceding works and the results of preliminary tests with Li_3_PO_4_, herein we present a detailed investigation of LNMFTO versus graphite full cells including Li_3_PO_4_ as cell additive, which allowed to further reduce the cell degradation (cf. blue circles in Figure 1).

In this manuscript, the issues related to cell aging in LNMO–graphite cells are addressed by the use of three approaches: a) use of an optimized Fe–Ti‐doped LNMO material with a rather low Mn^III^ content, b) reduction of metal bleeding by using a morphologically optimized active material with decreased specific surface area and c) capture of released metals (Mn, Ni) and HF by lithium phosphate.

## 2 Results and Discussion

### 2.1 Previous Results on LNMFTO and Li_3_PO_4_

The LNMFTO cathode active material investigated herein was synthesized in a two‐step spray drying approach, calcined under ambient atmosphere at 900 °C for 20 h and then held at 600 °C for 30 h. It corresponds to the starting material “A2,” from a previous study.^[^ ^14a^ ^]^ Briefly summarized, it consists of special granules with diameters of 6.3 µm (D10) to 14.3 µm (D90) with an average value of 10.1 µm. The specific surface area is ≈0.8 m^2^ g^−1^. The volume of pores within the granules was determined to be 20% by mercury intrusion porosity. The lattice parameter of LNMFTO (MgAl_2_O_4_ structure type, space group *Fd* $\bar{3}$ *m*) was refined to 818.61(3) pm. The secondary phase is Ni‐rich and has a trigonal crystal structure. Refinement as Li*_x_* Ni_1−_ *_x_* O_2_ (space group *R* $\bar{3}$m, *x* ≈ 0.33) yielded a phase fraction of 6.9 wt%. Taking into account Ni depletion of LNMO due to secondary phase formation, the results suggest the LNMFTO composition LiNi_0.47_Mn_1.40_Fe_0.1_Ti_0.03_O_3.95_ with a predominantly disordered arrangement of the transition metal cations within the octahedral framework of the spinel structure. In the preceding work^[^ ^14a^ ^]^ LNMFTO versus graphite cells already showed a superior cycling stability compared to other LNMO materials, as a capacity retention of 89% over 500 cycles at 0.5/0.5 C was observed.

**Figure 2:**
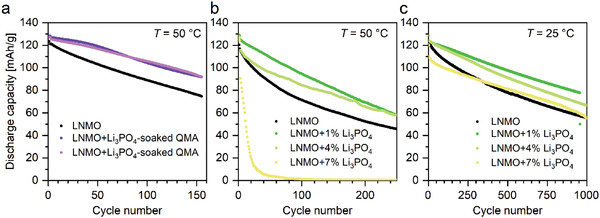
Preliminary cell tests with Li_3_PO_4_ as cell additive in LNMO versus graphite coin cells within a potential range between 3.5 and 5.0 V. Different cell aging of (a) LNMO and (c) LNMO results from different batches of material used. For these preliminary tests, deviating C rates of 0.5/0.5 (CC/CC) were used for charge and discharge cycles.

The detailed investigation of Li_3_PO_4_ as an additive in the case of LNMO cathode material was inspired by preliminary tests with impregnated separators, which were conducted with self‐made LNMO cathodes from a previous study.^[^ ^14b^ ^]^ These tests are depicted in **Figure** **2**. They showed that a significant improvement in cell aging could be achieved compared to nonadditivated cells at elevated temperatures of *T* = 50 °C (Figure 2a). Cells were cycled at current rates of 0.5 C during charging and 1.5 C during discharging, with current rates referenced to the theoretical capacity of 147 mAh g^−1^ of the LNMO material. However, impregnation of glass fiber separators is not a sui method to reasonably introduce additives into cells since 1) glass fiber separators basically have other properties than polyolefin or other commercially used separators, 2) they cannot be used in commercial cells, and 3) the amount of additive is quite difficult to control. Additionally, a new and challenging process step would be required, including incorporation of additives and drying steps. For this reason, attempts to directly incorporate Li_3_PO_4_ into the cathode material were made. For this purpose, different amounts of Li_3_PO_4_ were added to the slurry and the desired amount of Li_3_PO_4_ was adjusted directly during coating and production of the electrodes, respectively. Sufficient adhesion of the electrode material to the current collector was achieved. In the first step, a Mn^III^ rich LNMO material was used, with a proportion of active material in the layer between 86.2 and 92.6 wt%. Here, layers with 0–7 wt% Li_3_PO_4_ in the cathode material were produced. The results of the cell experiments are shown in Figure 2b at *T* = 50 °C and Figure 2c at *T* = 25 °C. The cells were built with commercial anode material (graphite) from Custom Cells, where the anodes were oversized in terms of area loading (factor of 1.2–1.6) and cycled up to a cutoff voltage of 5 V. It was observed that both at room temperature (*T* = 25 °C) and at elevated temperature of *T* = 50 °C, the cells containing Li_3_PO_4_ exhibited improved aging behavior compared to the Li_3_PO_4_ free standard cells. The higher temperature was chosen in order to observe aging effects more quickly (shortening the measurement time) and to detect effects of a varied temperature. It is noticeable, for example, that with an Li_3_PO_4_ content of 6.9% in the layer at 50 °C, very rapid aging (capacity reduction) occurs, whereas at *T* = 25 °C cycling over 1000 cycles is possible with 6.9% Li_3_PO_4_ additive (≈50% residual capacity). The most balanced and best properties in terms of capacity retention were obtained at a concentration around 1% Li_3_PO_4_. Based on these results, for the investigation of the Fe–Ti‐doped LNMO material LNMFTO, as the most promising approach, the admixture of 1–2 wt% Li_3_PO_4_ to the cathode layer was investigated further.

### 2.2 Electrode Properties and Li_3_PO_4_ Distribution

An overview of the LNMFTO cathodes investigated herein is given in **Table** **1**. Due to the manual processing and minor variations of the N–methyl–2–pyrrolidone (NMP) content in the slurries and small variations of the blade gap, the active materials loadings vary somewhat: For cathode A to F, the loadings were determined to be 10.3–12.0 mg cm^−2^. Based on the theoretical capacity of 147 mAh g^−1^, theoretical area capacities of 1.51–1.75 mAh cm^−2^ were calculated. Applying the experimentally accessible capacity of 130 mAh g^−1^, realistic areal capacities were estimated (marked by an*) to ≈1.34–1.55 mAh cm^−2^. The latter values were taken into account to calculate the anode‐to‐cathode balancing, which likewise is found in Table 1. The active mass of each cathode was determined by punching, weighing, and averaging at least six cathodes each.

**Table 1:** Cathode composition and properties

Cathode	Li_3_PO_4_	LNMFTO	Slit	Loading LNMFTO	Areal capacity	Balancing
	[wt%]	[wt%]	[µm]	[mg cm^−2^]	[mAh cm^−2^]	
A	0	90	200	11.3	1.67 (1.48*)	1:1.6
B	0	90	220	12.0	1.75 (1.55*)	1:1.5
C	1	89	200	10.4	1.53 (1.36*)	1:1.8
D	2	88	200	11.0	1.61 (1.43*)	1:1.7
E	0	90	200	10.9	1.61 (1.42*)	1:1.7
F	1	89	200	10.8	1.59 (1.40*)	1:1.7
G	1	89	300	17.3	2.54 (2.25*)	1:1.1

The two values of the specific area capacity refer to the theoretical capacity of 147 mAh g^−1^. Realistic area capacity values based on the 130 mAh g^−1^ are likewise listed (marked with an asterisk). The cell balancing (capacity of the cathode vs capacity of the anode) was calculated based on the realistic area capacities.

As a proof of principle that higher active material loadings can be applied without negative impacts on the coating process as well as the electrochemical performance, like, e.g., increased degradation or capacity loss, the active material loading of cathode G was substantially increased. With a theoretical capacity of 2.54 mAh cm^−2^, and realistic capacity of 2.25 mAh g^−1^ (based on the experimental capacity of 130 mAh g^−1^)^[^ ^14a^ ^]^ the cathode is balanced compared to the graphite anode (≈2.4 mAh cm^−2^). The thickness of the dried cathodes was between 110 and 100 µm, corresponding to overall porosities of 64% to 68%. With a calender heated to 50 °C, the cathodes were compressed to 60–70 µm, reducing the overall pore volume to 49%–55%.

Results of a more detailed investigation of cathode A are shown in **Table** **2**. It turns out, that the calendering step results in a strong reduction of the electrode specific resistance, which is the outcome of a combination of increasing the contact points within the electrode layer and—more importantly—decreasing the interface resistance between current collector and electrode layer. The adhesion strength benefits likewise from the enhanced contact zone to the Al foil. Due to the open pore structure of the LNMFTO secondary particles dissolved polyvinylidene difluoride (PVDF) binder is deposited also within the particles and is unable to contribute to adhesion (and cohesion) of the electrode layer. As a consequence, the adhesion strength values are on a lower level than what could be expected from compact active material particles.

**Table 2:** Cathode properties before and after calendering, investigated for cathode A, including the standard deviation of individual measurements

Cathode A	Before calendering	After calendering
Thickness [µm]	100 ± 5	71 ± 1
Porosity [%]	68 ± 3	55 ± 1
Specific resistance [Ω cm]	1100 ± 214	380 ± 62
Adhesive strength [N m^−1^]	15.0 ± 0.5	17.8 ± 0.7

The porosities were calculated based on the layer thickness, loading, composition, and the densities of the individual components.

**Figure 3:**
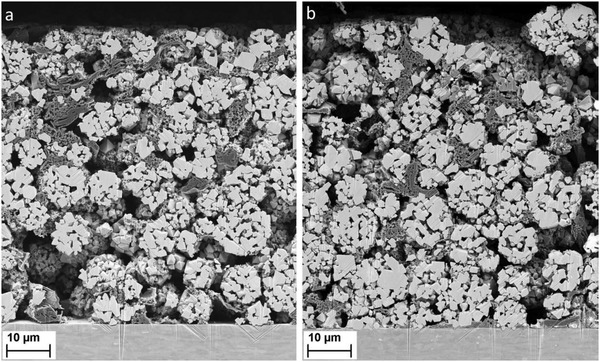
Cross section of cathode B before a) and after the 1029 cycle test program b). The coating layer has a thickness of ≈70 µm. The overall porosity is about 54%. Graphite is recognizable by its lamellar structure. Carbon black forms fine flakes. The cycling‐induced cracking in large LNMO particles becomes apparent only at higher magnification, corresponding images can be found in Figure S1 (Supporting Information).

A cross section image of cathode B is shown in **Figure** **3**. It reveals that the granules maintain their spherical form during calendering. Mechanical damage and cracking of LNMFTO particles is observed only in the uppermost region of the electrode, presumably as a direct result of the mechanical impact of the calendering.

**Figure 4:**
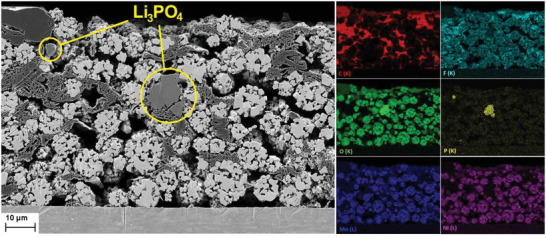
SEM/EDX investigation of cathode C prepared with 1 wt% Li_3_PO_4_. Based on the phosphorus signature (yellow), a larger, as well as some smaller pieces of Li_3_PO_4_ can be identified. Regions of the granules that are rich in secondary phases in particular can be recognized by a stronger Ni signature.

Results of a combined scanning electron microscope / energy–dispersive X–ray spectroscopy (SEM/EDX) investigation of a cross section of the uncycled cathode C containing 1 wt% Li_3_PO_4_ is shown in **Figure** **4**. The cathode thickness is ≈65 µm. Within the active material granules, small areas with increased Ni concentrations become visible and indicated the presence of Ni‐rich secondary phase. Li_3_PO_4_ grains clearly can be distinguished from the rest of the electrode on the basis of the phosphorus and oxygen signatures in the EDX maps. The images confirm that lithium phosphate is embedded in the matrix formed by carbon black, graphite, and PVDF. The single grains of Li_3_PO_4_ found at different locations of the cross sections have diameters of 1–15 µm.

A confirmation and quantification of crystalline Li_3_PO_4_ in the electrodes was achieved by means of powder X‐ray diffraction (PXRD). The diffraction pattern of cathode C containing 1.0 wt% Li_3_PO_4_ was measured in reflection geometry and refined by the Rietveld method. A detailed discussion is presented in Section 2.4.

### 2.3 Cell Tests with LNMFTO and Li_3_PO_4_ Additive

**Figure 5:**
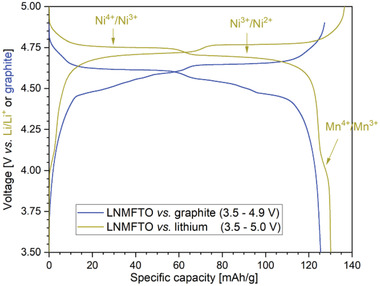
Comparisons of the voltage profiles of the second cycles for LNMFTO versus Li anode (yellow) and LNMFTO versus Graphite anode (blue) at a charge/discharge rate of 0.1 C.

When cycled against a lithium anode, for LNMFTO cathode A, a discharge capacity of 130.1 mAh g^−1^ was obtained in the second cycle (0.1 C/0.1 C for charge/discharge). As discussed previously,^[^ ^14a^ ^]^ and as displayed in **Figure** **5** (yellow profile) this capacity mainly can be assigned to Ni^4+^/Ni^3+^ and Ni^3+^/Ni^2+^ redox activity (≈124 mAh g^−1^, 95%). A minor contribution originates from the Mn^3+/4+^ redox couple (≈6 mAh g^−1^, 5%).

The voltage profile displayed in blue was obtained by cycling the same cathode against a graphite anode. The reduction of the cathode potential by roughly 0.1–0.2 V compared to the cell cycled against Li is a common feature of cells cycled against graphite. Supplementary investigations were carried out with a three electrode setup. The results are consistent with findings and conclusions from Asenbauer et al.^[^ ^19^ ^]^ and can be found in Figure S2 in the Supporting Information. Apart from the reduced potential of the LNMFTO versus graphite cell, compared to the LNMFTO versus Li cell, the specific capacity of the second cycle is reduced by roughly 4.7–125.4 mAh g^−1^. We assume that these losses are assigned to the formation of the solid electrolyte interface (SEI), roughly according to Equation (1)

(1) $$\begin{array}{ccc} & & {\mathrm{LiNi}}_{0.47}^{\mathrm{II}}{\mathrm{Mn}}_{1.35}^{\mathrm{IV}}{\mathrm{Mn}}_{0.05}^{\mathrm{III}}{\mathrm{Fe}}_{0.1}{\mathrm{Ti}}_{0.03}O_{3.95}\overset{-0.04\mathrm{Li}}{\underset{\mathrm{SEI}\;\mathrm{formation}}{\to }}{\mathrm{Li}}_{0.96}\\ & & \;{\mathrm{Ni}}_{0.47}^{\mathrm{II}}{\mathrm{Mn}}_{1.39}^{\mathrm{IV}}{\mathrm{Mn}}_{0.01}^{\mathrm{III}}{\mathrm{Fe}}_{0.1}{\mathrm{Ti}}_{0.03}O_{3.95} \end{array}$$

Initial Li loss thus leads to an irreversible oxidation of Mn^III^ to Mn^IV^ and drastic reduction of manganese related redox activity. The Mn^III/IV^ step, which is clearly visible in the voltage profile of the half‐cell, is not recognizable in the full cell. Continuing LNMFTO redox activity thus can almost fully be assigned to the Ni^II/III^ and Ni^III/IV^ redox couples and it seems plausible, that this has a beneficial impact on cycling stability, as Mn^III^ is considered to play a central role in degradation of LNMO–graphite full cells. Generally speaking, this means that for undoped LNMO materials with a formation related Li loss of 0.06 (6%), for the spinel phase LiNi_0.5−_ *_x_* Mn^IV^_1.5−_ *_x_* Mn^III^_2_ *_x_* O_4_, *x* should not exceed 0.03. For a detailed discussion of the composition of the spinel phase, the reader is referred to reference.^[^ ^7^ ^]^ From this perspective, however, it seems that virtually all LNMO materials used in previous studies on full cells had Mn^III^ contents that were unfavorably high.

**Figure 6:**
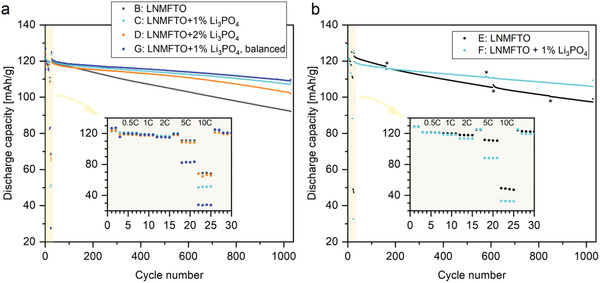
Cell aging of LNMFTO versus Graphite full cells with and without Li_3_PO_4_ as cell additive cycled in a voltage range between 3.5 and 4.9 V. a) CR2032 coin cells cycled at 26 °C. b) Pouch bag cells cycled at 23 °C (* discontinuities in capacity result from temporary interruptions in cell testing due to power outages). Detailed information on the individual properties of cathodes B to F and on the C rates during the cell tests is given in Tables 1 and S3, respectively.

Based on the preliminary tests from Section 2.1, the positive properties from LNMFTO developments were combined with the promising additive features of Li_3_PO_4_. For this purpose, LNMFTO was used to prepare cathodes B, C, and D containing 0, 1, and 2 wt% Li_3_PO_4_. The cycling of the cells is shown in **Figure** **6**. An overview of all cell tests with an assignment of the cathodes to the figures as well as central test results can be found in Table S2 in the Supporting Information. For the test of the additive concentrations, the sizing between anode and cathode was still in the order of 1.5–1.8:1 (real capacity of anode to cathode, for details see Table 1). 1000 cycle capacity retentions of 76.9%, 88.7%, and 85.9% were obtained. The latter refer to the 1000 cycles with constant current constant voltage (CCCV) charge and constant current (CC) discharge mode. At this stage, the sizing between cathode and anode was also adjusted. Cathode G with 1% Li_3_PO_4_ and an active material loading of 17.3 mg cm^−2^ was prepared so that self‐manufactured anode layer was used with an overdimensioning of the anode of 1:1.1 (real capacity of cathode to anode). The corresponding cell test is labeled as “balanced” in Figure 6a. In this case, a capacity retention of 90.2% was received over 1000 cycles at *T* = 26 °C in a potential range of *U* = 3.5–4.9 V. The cycling was also conducted with pouch bag cells, to demonstrate the proof of concept of the ability to constructs larger cells under dry room conditions. In Figure 6b, pouch bag cells made from cathodes E and F with capacities of 35.0 and 35.5 mAh (0.1 C) are shown. Capacity retentions of 79.2% and 88.5% over 1000 cycles at *T* = 23 °C in a potential range between 3.5 and 4.9 V were obtained. We found no evidence for gas formation in the LNMO versus graphite pouch bag cells. Our results seem to confirm the findings of R. Jung et al.,^[^ ^11^ ^]^ which showed, that at room temperature, LNMO does not promote chemical oxidation of the electrolyte at voltages <4.9 V.

After cell formation, a short rate capability test was performed and results are shown in the enlarged section in Figure 6. It becomes obvious, that there are only minor discharge capacity losses at discharge rates up to 5 C. Fast discharging with 10 C leads to a significant loss of the capacity, which however might well be related to the graphite anode (cf., e.g.,^[^ ^20^ ^]^ since the effect was not observed when LNMFTO was cycled versus a Li anode.^[^ ^14a^ ^]^

**Figure 7:**
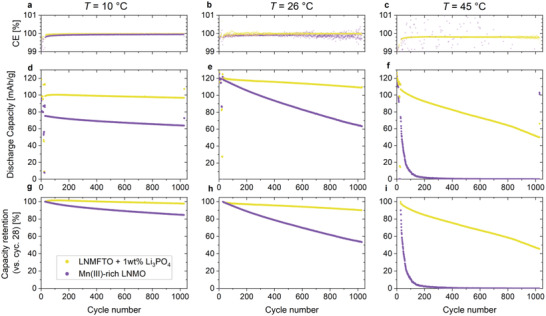
LNMFTO + 1% Li_3_PO_4_ (cathode G, yellow) and a Mn^III^‐rich LNMO material (violet) cycled in CR 2032 coin cells versus graphite anodes at 10, 25, and 45 °C.

A major obstacle for LNMO–graphite full cells used to be the inferior cycling stabilities at elevated temperatures. In order to investigate the impact of different temperatures on the cycling behavior of the optimized Li_3_PO_4_ containing LNMFTO cathode G, further CR2032 full cells were built and cycled at 10 and 45 °C (**Figure** **7**). Figure 7b shows a comparison of the results with the previous tests performed at room temperature. The standard testing protocol specified in Table S3 (Supporting Information) was used, and in order to have some reference data, residual LNMO cathodes from a previous study^[^ ^14b^ ^]^ were investigated accordingly. The latter contain a rather Mn^III^ rich LNMO phase (≈LiNi_0.45_Mn_1.55_O_4_ = LiNi^II^_0.45_Mn^IV^_1.45_Mn^III^_0.1_O_4_), have an areal capacity of ≈1.5 mAh cm^−2^ and a composition of 88/4/4/4 (LNMO/graphite/carbon black/PVDF, wt%). As a result of the lower active mass loading, the cells are not as well balanced as the LNMFTO cells. Hence, we will not discuss these cells in detail, but we consider the comparative data as useful.

As shown in **Table** **3**, the CE's of the formation cycles are reduced for the cells cycled at 10 °C. The low values, especially for the Mn^III^‐rich LNMO, could be an indication that SEI formation at 10 °C is more Li‐consuming compared to 26 and 45 °C. During the cycling stability test (cycle 28 to cycle 1027), the Coulombic efficiencies obtained for the cells with LNMFTO + 1 wt% Li_3_PO_4_ in contrast are above 99.97 for 10 and 25 °C, respectively. At 25 °C, the individual CE values scatter significantly, probably due to less accurate hardware and due to slight temperature fluctuations in the measurement rooms. At 45 °C the CE is reduced to roughly 99.8%, presumably as a direct result of Li loss in each cycle.

**Table 3:** Coulombic efficiencies and charge/discharge capacities of the full cells during the first two cycles for different temperatures (charge and discharge rates: 0.1 C)

Cathode	Cycle	10 °C			26 °C			45 °C		
		C^a)^	DC^a)^	CE	C^a)^	DC^a)^	CE	C^a)^	DC^a)^	CE
		Capacity [mAh g^−1^]		[%]	Capacity [mAh g^−1^]		[%]	Capacity [mAh g^−1^]		[%]
G: LNMFTO + 1 wt% Li_3_PO_4_	1	134.0	118.8	88.7	138.6	126.8	91.5	144.9	126.7	87.4
	2	120.4	118.5	98.4	128.8	127.3	98.8	130.3	123.6	94.9
Ref: Mn^III^‐rich LNMO	1	137.1	94.1	68.6	140.7	125.5	89.2	145.1	123.9	85.4
	2	95.9	91.4	95.3	127.3	125.3	98.4	127.5	122.0	95.7

^a)^ C: charge; DC: discharge.

**Figure 8:**
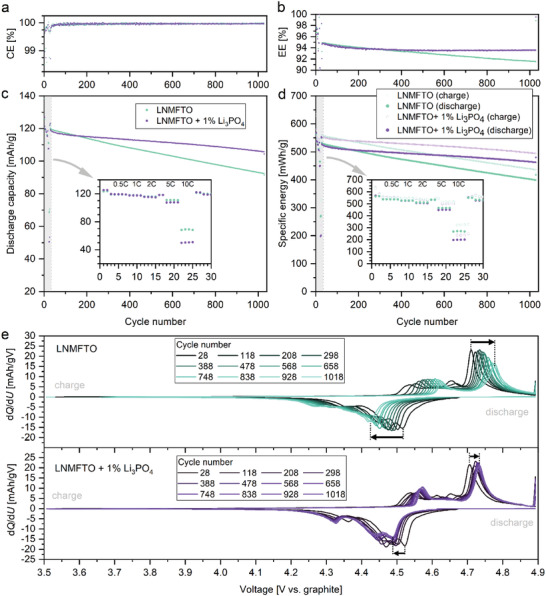
Comparison of LNMFTO (turquoise) and LNMFTO +1% Li_3_PO_4_ (violet) cathodes E and F cycled against graphite anodes. a) Coulombic efficiency (CE). b) Energy efficiency (EE) (cathode active material mass only). c) Discharge capacity versus cycle number. d) Specific energy versus cycle number. Light and dark color tones correspond to charging and discharging, respectively. e) d*Q*/d*U* curves in steps of 90 cycles during the cycling stability test (CCCV charging, 1 C; CC discharging, 1.5 C).

It becomes obvious that the LNMFTO + 1 wt% Li_3_PO_4_ cathodes are superior to the Mn^III^‐rich LNMO material at all temperatures. For the cells built with Cathode G during the cycling stability test, a minor capacity loss is observed at 10 °C. Over 1000 cycles, a capacity retention of 97.8% is found and the residual capacity is 96.8 mAh g^−1^. In contrast, at 45 °C, the 1000 cycle capacity retention is reduced to 45.6% (residual capacity: 49.8 mAh g^−1^). As discussed above, at 26 °C, a capacity retention of 90.2% over 1000 cycles was obtained, for the LNMFTO material. The most drastic between LNMFTO and the Mn^III^ rich LNMO material is observed at 45 °C, where the latter quickly fails. However, from the capacity of “cycles” 1028 and 1029 and a closer inspection of the voltage profiles (not shown) it becomes obvious that this failure is related to strong cell polarization. After roughly 200 cycles, the voltage window of 3.5–4.9 V no longer covers the charge and discharge processes and hence, the cells are no longer able to provide noteworthy discharge capacity. Hence, this type of degradation is not related to Li loss, as it is the case for the corresponding cell containing LNMFTO + Li_3_PO_4_. Based on the results of the initial concentration test, new LNMFTO cathodes E and F containing 0 and 1 wt% Li_3_PO_4_ were manufactured and cycled for a more detailed investigation of the effect of Li_3_PO_4_ (**Figure** **8**).

During cell formation, coulomb efficiencies (CE) between 85% and 90% in the first cycle as well as 98% and 99% for the second cycle were found, which then increased to >99.5% in the fourth cycle and then remained stable at >99.9% during the cycling stability test. The specific charge and discharge energies for the cells and the resulting energy efficiencies (EE) are shown in Figure 6b. After the formation cycles and the rate capability test, energy efficiencies around 98.5% were found 27th cycle charge and discharge rates of 0.2 C. This indicates that energy losses due to the open circuit voltage (OVC) hysteresis should be 1.5% or below and apparently, the OCV hysteresis hardly increases during cycling, as we find energy efficiencies of 98% and above when performing 0.2 C cycling again after the 1000 cycles of the stability test.

The energy loss in the LNMFTO graphite cells thus predominantly is a result of increasing cell polarization with higher currents and cycle numbers. The current dependent polarization becomes obvious from the initial rate capability test. With increasing discharge rates, the energy efficiency decreases according to ≈97.5%, 96.5%, 95%, 90%, and 85% for 0.5 C, 1 C 2 C, 5 C, and 10 C. A positive effect of Li_3_PO_4_ is not detectable at this early stage of battery cell testing. However, when it comes cycle related cell polarization, the decisive advantage of the Li_3_PO_4_ cell additive becomes evident. As shown in Figure 8e, the cell polarization almost linearly increases in the LNMFTO graphite cell. Following the maxima of the d*Q*/d*U* plots, a voltage polarization accounts to 0.20 V in the 28th cycle and increases to 0.24 and 0.36 V over the next 200 and 1000 cycles, respectively. In the Li_3_PO_4_ containing cell, the voltage polarization is ≈0.19 V in the 28th cycle. It increases to 0.23 V after 200 cycles. After roughly 300 circles a polarization of 0.24 V is reached, which remains rather stable. As a result, over the 1000 cycle stability test, energy efficiency of the LNMFTO cell constantly decreases from 94.8% to 91.5%. The EE of the Li_3_PO_4_ containing cell similarly fades from 94.7% to 93.6% over the first 300 cycles, but thereafter it remains stable at high levels > 93.5%.

**Figure 9:**
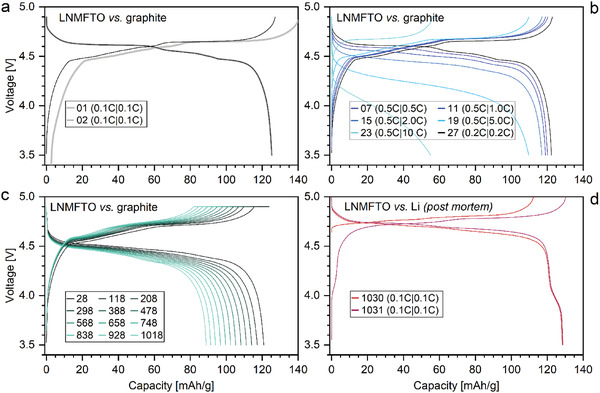
Voltage profiles of a LNMFTO versus graphite coin cell (cathode E). a) Formation cycles, b) rate capability test, and c) cycling stability test (CCCV charge, 1 C discharge). d) Postmortem cycling of the same cathode versus fresh Li.

**Figure** **9** shows representative voltage profiles of a LNMFTO (cathode E) versus graphite full cell during the formation cycles (subfigure (a)), the rate capability test (b) and the cycling stability test (c). In the voltage range of 3.5–4.2 V, no redox plateau can be detected. This indicates that, as mentioned at the beginning, manganese related redox activity is avoided effectively and manganese prevails in the oxidation state IV. Thus, during charging and discharging, nickel is oxidized and reduced exclusively. Even after >1000 cycles, the major part of the discharge capacity is found above 4.3 V at a discharge rate of 1.5 C.

### 2.4 Postmortem Analysis

To gain a deeper understanding of the impact of Li_3_PO_4_ and the cell degradation mechanisms, comprehensive postmortem investigations were carried out by SEM/EDX, PXRD, inductively coupled plasma optical emission spectrometry (ICP‐OES), and reuse of cycled LNMFTO cathodes versus Li metal anodes.

SEM/EDX investigation of cycled cathodes showed that after 1029 cycles, no more distinct Li_3_PO_4_ particles were detectable in the cross‐sections. Although the presence of phosphor was confirmed by EDX analysis, it has to be kept in mind that reaction products of LiPF_6_ from the electrolyte are another potential source of P. Apart from the missing Li_3_PO_4_, the structure of the cathodes was largely intact. Individual cracks were visible, especially in large LNMFTO particles, but no significant deterioration of the active material or the electrode was observed (see Figure 3b and Figure S1 in the Supporting Information).

**Figure 10:**
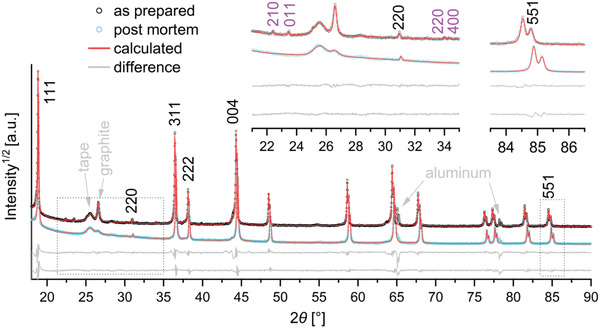
X‐ray diffraction patterns of the LNMFTO cathode C prepared with 1 wt% Li_3_PO_4_ before (black) and after cycling (cyan). Reflection indices for LNMFTO and Li_3_PO_4_ are shown in black and purple. The sections of 23° < 2*θ* < 35° and 83° < 2*θ* < 87° show the main reflections of *γ*‐Li_3_PO_4_ and highlight the shift of the LNMFTO reflections due to a decreasing lattice parameter. The square root of the intensity was chosen to emphasize the weak reflections.

An extracted plate of the cycled cathode C (prepared with 1.0% Li_3_PO_4_) and a plate of the uncycled cathode were investigated by means of PXRD. The diffraction patterns were evaluated by the Rietveld method and compared, the results are shown in **Figure** **10**. Both diffraction patterns show the typical reflections related to the LNMFTO spinel phase (black indices) with reflection shoulders related to the Li*_x_* Ni_1−_ *_x_* O secondary phase (refined as Li_0.33_Ni_0.66_O), for instance at 2*θ* ≈ 44° and 2*θ* ≈ 64°. Additionally, the graphite 002 reflections (2*θ* ≈ 27°) from the conductive additives are visible. Further scattering contributions from the aluminum current collector (2*θ* ≈ 65° and 2*θ* ≈ 77°, not refined) and the adhesive tape used to fix the cathode plates (2*θ* ≈ 25.5° and 2*θ* < 18°, not shown) are observed in both patterns. In the uncycled cathode, additional reflections at 2*θ* = 22.4°, 23.5°, 34.0°, and 34.3° are observed, which could be refined using a structure model of *γ*‐Li_3_PO_4_ (space group: *Pnma*),^[^ ^21^ ^]^ yielding suitable lattice parameters of *a* = 1046 pm, *b* = 612 pm and *c* = 485 pm. The phase fraction of Li_3_PO_4_ was refined to be 0.9(2) wt% and thus perfectly agrees with the 1 wt% expected. Li_3_PO_4_ reflections are observed exclusively in the uncycled cathode, after cycling, Li_3_PO_4_ reflections can no longer be recognized, confirming the dissolution of the phase during cycling.

For LNMFTO in the uncycled cathode, a lattice parameter of 818.3(2) pm was obtained, which is very close to the 818.61(3) pm refined for an LNMFTO pattern measured with an advanced in‐house PXRD setup that uses transmission geometry.^[^ ^14^, ^22^ ^]^ An interesting detail is the reduction of the LNMFTO lattice parameter in the cycled cathode. The postmortem cathode was extracted from the cell after slow charging (0.2 C) and thus should contain *all* lithium electrochemically active after 1029 cycles. While the lattice parameter was determined to be 818.3(2) pm in the uncycled cathode, for the postmortem cathode the refinement yields a reduced lattice parameter of 815.5(2) pm. It is known from in‐operando XRD studies, that disordered LNMO materials show solid solution like behavior for 0 < *x* < 0.5 in Li_1−_ *_x_* Ni_0.5_Mn_1.5_O_4_.^[^ ^23^ ^]^ This means, that for highly lithiated states, the lattice parameter decreases almost linearly with the degree of delithiation. Accordingly, based on a study of Kunduraci,^[^ ^23^ ^]^ a lattice parameter reduction of 2.8 pm can be estimated to correspond to *x* = 0.16 for Li_1−_ *_x_* Ni_0.5_Mn_1.37_Fe_0.1_Ti_0.03_O_3.95_, corresponding to a loss of 16% of cyclable lithium during the cell test.

Hence, the decrease of the lattice parameter due to cycling can be used as a parameter for the loss of cyclable Li in the full cell. As reported before,^[^ ^13f^ ^]^ lithium loss should be considered as the main reason for the capacity loss in the LNMFTO versus graphite cells presented herein. This assumption is further supported by the cycling behavior of the “aged” cathodes (see below).

A cathode E plate extracted from a cycled coin cell was washed with dimethyl carbonate (DMC), dried, and cycled again with a fresh lithium anode in a new coin cell. Voltage–capacity profiles from the postmortem cycling are shown in Figure 9d. The discharge capacity in the full cell decreased from 125.4 mAh g^−1^ (second cycle, 0.1 C) to 88.6 mAh g^−1^ in cycle 1029 (0.2 C). Afterward a discharge capacity of 128.7 mAh g^−1^ was found when using fresh lithium with the cycled electrodes. Similar capacities were reported for Fe–Ti‐doped LNMO previously^[^ ^14a^ ^]^ and highlight, that the LNMFTO material itself does hardly take any damage from cycling. As soon as enough lithium is available, the full discharge capacity is reached again. The reduced charge capacity of the first postmortem cycle (112.3 mAh g^−1^) compared to the second postmortem cycle (130.3 mAh g^−1^) is a result of the Li loss in the full cell. Lithium is extracted during charging, and starting from a reduced Li content, with for instance *x* = 0.3 in Li_1−_ *_x_* Ni_0.47_Mn_1.40_Fe_0.1_Ti_0.03_O_3.95_ limits the charge capacity. The following discharging allows for full relithiation of LNMFTO (*x* ≈ 0), and finally, in the second post mortem cycle the full charge capacity is observed.

Based on the residual capacity of 88.6 mAh g^−1^ in the full cell and the postmortem half‐cell capacity of 128.7 mAh g^−1^, the overall lithium losses of the full cell, which is shown in Figure 9a–c, can be estimated to be 31% over the 1029 cycles. Accordingly, an end of life spinel phase composition Li_0.69_Ni_0.47_Mn_1.40_Fe_0.1_Ti_0.03_O_3.95_ can be expected in the fully charged state. In the best cells built from the Li_3_PO_4_ containing cathode G, residual capacities around 109 mAh g^−1^ were observed. Accordingly, the total lithium loss can be estimated to be 15% over 1029 cycles, which corresponds to an end of life composition of Li_0.85_Ni_0.47_Mn_1.40_Fe_0.1_Ti_0.03_O_3.95_ in the charged state. These results are thus fully consistent with the findings on the Li loss from the PXRD investigation.

**Figure 11:**
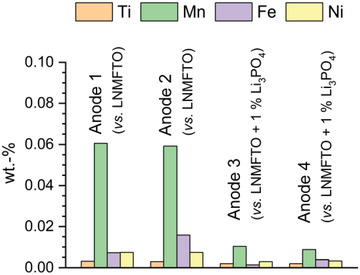
Results of the ICP‐OES analysis of four graphite anodes extracted from CR2032 coin cells after 1029 cycles. Anodes 1 and 2 were cycled against cathode E (LNMFTO). Anodes 3 and 4 were cycled against Cathode F (LNMFTO + 1 wt% Li_3_PO_4_).

Regarding the transition metal or phosphorus content of the cycled anodes, no meaningful results could be obtained by SEM/EDX. By means of PXRD, no differences were detected either, which is why investigations were carried out using ICP‐OES. Therefore, the transition metal content of two graphite anodes cycled against cathode E (LNMFTO) and of two anodes cycled against cathode F (LNMFTO + 1 wt% Li_3_PO_4_) were investigated. All cells completed the same battery test program of 1029 full cycles as described in Table S3 (Supporting Information). The results of the investigation are depicted in **Figure** **11** and Table S4 (Supporting Information). As reported in previous studies,^[^ ^15c^ ^]^ it becomes obvious, that manganese in particular migrates from the cathode side to the anode, however, ICP‐OES also proves the deposition some Ni, Fe, and Ti during cycling.

As expected, the anodes cycled against the same cathodes show similar results. For anodes 1 and 2, extracted from cells without Li_3_PO_4_, the atomic ratio of Ni:Mn is 1:8.7 which is significantly higher than in LNMFTO (1:2.7). Such ratios prove a preferential dissolution, transport and decomposition of manganese compared to nickel. Iron likewise seems to be overrepresented on the anode side with a Ni:Fe ratio of 1:1.6, compared to LNMFTO (1:0.2). However, the iron contents of the anodes differ somewhat, and the coin cell cases cannot be excluded as a possible iron source, so that further investigations are necessary for a more reliable quantification of Fe dissolution from LNMFTO.

Nevertheless, anodes 3 and 4 cycled against cathode F containing 1 wt% Li_3_PO_4_ show remarkable reductions of the overall transition metal content by 79% compared to anodes 1 and 2. The individual reductions vary somewhat. Mn and Fe content is reduced drastically by 84% and roughly 77%. The content of Ni and Ti is reduced by ≈57% and 36%. ICP‐OES investigations thus show that the cathode‐additive Li_3_PO_4_ effectively minimizes transition metal deposition on the anode side.

### 2.5 The Effect of Li_3_PO_4_

Li_3_PO_4_ does not seem to increase the starting capacity of LNMFTO materials, and thus, in contrast to other solid additives like, e.g., lithium oxalate that decomposes during cell formation,^[^ ^13m^ ^]^ the mechanism is more complex than just adding lithium to the system. Compared to the impact of Li_3_PO_4_, the additional amount of Li is actually quite small. In the coin cells investigated herein, 23 mg LNMFTO, 0.23 mg Li_3_PO_4_ and 110 µL LP30 are present. This means that the lithium originating from 1 wt% Li_3_PO_4_ provides only about 5% additional lithium compared to the similar amounts provided by LNMFTO or LiPF_6_. Based on our observation, Li_3_PO_4_ is not easily soluble in the LP30 electrolyte; however, it appears that at least small portions of Li_3_PO_4_ will dissolve in equilibrium reactions, and as soon as dissolved species react, the equilibria will shift further, leading to a slow dissolution of all Li_3_PO_4_ according to Equation (2)

(2) $$\begin{array}{ccc} {\mathrm{Li}}_{3}{\mathrm{PO}}_{4}\left(\mathrm{solid}\right) & ⇌ & {\mathrm{Li}}_{2}{\mathrm{PO}}_{4}^{-}\left(\mathrm{solv}.\right)+{\mathrm{Li}}^{+}\left(\mathrm{solv}.\right)\\ & & ⇌{\mathrm{LiPO}}_{4}{{}^{2}}^{-}\left(\mathrm{solv}.\right)+2\;{\mathrm{Li}}^{+}\left(\mathrm{solv}.\right) \end{array}$$

This assumption is supported by the XRD investigations and the results of SEM/EDX investigation, where after cycling no local phosphorus accumulations were detectable on the cathode side. It is also well conceivable that Li_3_PO_4_ or the corresponding anions like Li_2_PO_4_^−^ function as proton scavengers and thus suppresses the parasitic reaction between H^+^ ions and LiPF_6_ with the formation of HF, e.g., according to Equation (3)

(3) $${\mathrm{Li}}_{2}{\mathrm{PO}}_{4}^{-}\left(\mathrm{solv}.\right)+H^{+}\left(\mathrm{solv}.\right)\to {\mathrm{Li}}_{2}{\mathrm{HPO}}_{4}↓$$

Similar HF scavenging reactions of additives have been reported recently by the Lucht Group.^[^ ^24^ ^]^ A reduction of the acid‐mediated transition‐metal ion dissolution and related shuttle or crossover reactions was described and considered the main reason for increased cycling stability in LNMO Graphite cells. For example, lithium bis(trimethylsilyl) phosphate (LiTMSP), which is reported to bind HF, is described by Kim et al. to enhance aging in the case of LNMO/graphite full cells.^[^ ^24b^ ^]^ However, since a significant decrease in capacity was already observed within 100 cycles, it is difficult to estimate the actual contribution of LiTMSP to the improvement.^[^ ^24b^ ^]^

In the present study, cells were extracted with water (20 mL for each pouch bag cell) after cycling and the pH of the aqueous solution was measured directly after the treatment. It was shown that the cells without Li_3_PO_4_ had slightly lower pH values (pH = 7.19–7.24) compared to the cells with Li_3_PO_4_ (pH = 7.30–7.35). However, since the changes were very small, this is only an indication of a possible influence of the Li_3_PO_4_ additive with respect to the acid value in the cell.

In addition, based on the drastically reduced transition metal content on the anode side, it could also be assumed that Li_3_PO_4_ or its anions are able to react with transition metal ions directly. In this manner, immobilization of these transition metal ions may be accomplished. Numerous stable phosphates *M_x_* (PO_4_)*_y_* or Li*_x_M_y_* (PO_4_)*_z_* (*M* = Mn, Ni, Fe, Ti) are known and reactions, e.g., according to Equation (4) could occur, with the consequence, that dissoluted transition metal ions no longer trigger SEI and cell deterioration. However, within this study we were not able to identify LiMnPO_4_ with SEM/EDX or other methods used here

(4) $${\mathrm{LiPO}}_{4}{{}^{2}}^{-}+{\mathrm{Mn}}^{2+}\left(\mathrm{solv}.\right)\to {\mathrm{MnLiPO}}_{4}↓$$

Based on these hypothesis on the effect of Li_3_PO_4_ in full cells, similar reactions are to be expected for deviating cell chemistries. And indeed, several studies report positive effects of Li_3_PO_4_ on the cycling stability of state of the art Ni‐rich layered oxide cathode materials like LiNi_0.8_Co_0.15_Al_0.05_O_2_^[^ ^25^ ^]^ or LiNi_0.83_Co_0.11_Mn_0.06_O_2_.^[^ ^26^ ^]^ We therefore assume that our approach based on the admixture of crystalline Li_3_PO_4_ to cathode slurries should yield similar positive effects. However, we would like to emphasize that for all reactions discussed experimental validation is needed and most likely, more sophisticated methods and experimental approaches are required.^[^ ^15c^ ^]^

## 3 Conclusion

Improvements of the cycling stability of Li_3_PO_4_ coated LNMO compared to bare LNMO cycled against graphite anodes have been already reported. However, our results clearly proof that a random distribution of 1 wt% crystalline Li_3_PO_4_ within the cathode layer can have a significant impact on the cycling stability and thus, homogeneous surface coatings are not mandatory at all. As a result, following our Li_3_PO_4_ admixing approach, large‐scale material and cathode processing could be kept simple and cost efficient.

Our findings clearly confirm that LNMO versus graphite cells can be reliably cycled with standard carbonate‐based electrolytes at voltages to 4.9 V, including CV charging steps. A prerequisite for achieving this apparently is, that the amount of Mn^III^ in the LNMO phase should not exceed the amount of lithium which is lost during SEI formation. If this principle is met, manganese largely remains in the oxidation state Mn^IV^ and dissolution is partly inhibited. Indications for electrolyte oxidation were not observed. Another central problem of LNMO versus graphite cells, namely the loading of the cathode layers has likewise successfully been addressed in this work. The porous structure of the LNMFTO granules allow for high cathode loadings and in fact, the best cycling stability with a remaining capacity of 109.9 mAh g^−1^ related to the active material after 1030 cycles was obtained for a cathode with an active material loading of 17.3 mg cm^−2^, which corresponds to a capacity of >2.2 mAh cm^−2^. The corresponding 1000 cycle capacity retention was determined to be 90.2%. It was surely not the aim of the present study to determine the limitations of the active material loadings, but we see no reason why the active material loadings could not be increased even further.

Our results prove that the crystalline Li_3_PO_4_ is dissolved after cycling, and that chemical cross talk, i.e., the decomposition of transition metal ions on the anode side was reduced by roughly 80%. Manganese deposition on the graphite anode, which is considered as the main cause of cell ageing, was reduced even by 84%. The exact mechanisms and reactions leading to the beneficial influence of Li_3_PO_4_, however, are difficult to determine and still pending questions.

The cells containing Li_3_PO_4_ do still suffer from some Li‐loss, which was confirmed as predominant source of capacity fade. However, after initial increase, the cell polarization of Li_3_PO_4_ containing full cells ceased after roughly 300 cycles, indicating a very stable SEI, and thus, cycling over several thousand cycles is coming into reach. This was achieved without any further additives in the electrolyte. The finding that the LNMFTO cathodes suffer practically no damage and regain their full capacity as soon as sufficient lithium is available also supports this assumption. Upcoming engineering challenges are the use of thin polyolefin‐based separators and the reduction of the amount of electrolyte.

## 4 Experimental Section

### Material Preparation

The LNMFTO cathode active material was prepared in a two‐step spray‐drying process, starting from the acetates of Li, Ni and Mn, iron nitrate and tetra‐*iso*‐propyl *ortho*‐titanate. After dissolution in water, stoichiometric ratios of the aqueous solutions were mixed and spray dried. The powder obtained was calcined for 2 h at 500 °C, cooled to room temperature and calcined again at 800 °C (2 h). After mixing with distilled water, the precursor was ground for 24 h in a planetary ball mill (3 mm ZrO_2_ grinding balls) and spray dried again. From a final temperature treatment at 900 °C (20 h) followed by an annealing step at 600 °C (30 h) the LNMFTO active material was obtained. A detailed description is found in the previous publication.^[^ ^7^ ^]^ Comprehensive data about auxiliary compounds of the cathode and anode layers are presented in Table S1 (Supporting Information). Cathodes with a final ratio of 90/3/3/4 wt% (active material/carbon black/graphite/PVDF) were prepared by mixing the LNMFTO, with previously prepared NMP‐based slurries containing carbon black (C‐NERGYSUPER C65, Imerys), graphite (ABG1010, Superior Graphite), and PVDF (Solef 5130, Solvay). For the lithium phosphate containing cathodes, based on the mass of LNMFTO, 1 or 2 wt% Li_3_PO_4_ (ABCR, >98%) was added to the mixture, leading to slightly deviating compositions of ≈89/3/3/4/1 or 88/3/3/4/2. Both Li_3_PO_4_ and LNMFTO were sieved with a 50 µm mesh prior to addition to the slurry. After mixing, the slurries were coated on aluminum foil using a blade with a slit of usually 200 µm and dried to 80 °C for one hour on the film application device (Coatmaster 510, Erichsen). In the next step, cathodes were transferred into a vacuum chamber and dried at 110 °C for at least 14 h. After drying, the electrodes were calendered at 50 °C (details below). To facilitate and improve readability and clarity, Table 1 provides an overview of the cathodes used. Another table with an assignment of the cathodes to individual figures as well as central results of the cell tests can be found in Table S2 in the Supporting Information. Graphite anodes were manufactured by an aqueous processing route. Graphite (96 wt%, Hitachi SMG‐A) with an average particle size of about 20 µm was used as the active material. Carbon black (1.5 wt%, C‐NERGYSUPER C65, Imerys) was applied for improving binder conductivity. A solution of 1.25 wt% sodium carboxymethyl cellulose (WALOCEL CRT 2000 PA07, DOW) in deionized water was used as one binder component. Styrene‐butadiene rubber (1.25 wt%, TRD2001, JSR Micro) supplied as a latex binder with 170 nm particle size served as the second binder. After coating on Cu foil and drying, the anodes were calendered to a porosity of ≈50%. The capacity of the anode was calculated to be 2.4 mAh cm^−2^. For CR2032‐type coin cells, SUS316L stainless‐steel cans (Hohsen, Japan) were used. The dried electrodes were transferred to an argon filled glovebox under inert conditions. LNMFTO cathodes with diameters of 15 mm were used. As separator, Whatmann QMA was deployed (*Ø* = 17 mm). The graphite anodes had diameters of 15 or 16 mm. In each cell, 110 µL 1 M LiPF_6_ in ethylene carbonate /DMC electrolyte (LP30, Sigma Aldrich) was used. Pouch cells were assembled in a dry room with a dew point below −70 °C. Cathodes and anodes with 50 and 52 mm edge length were used. As separators, commercial polyethylene terephthalate‐based particle filled separator (cathode E) and Whatman QMA (cathode F) were deployed and likewise wetted with LP30 Electrolyte (450 and 1.5 mL). A gas trap was inserted before vacuum sealing the pouch bag cells to ensure a stable vacuum inside the cell after formation. No additional reopen/closing procedures were used (e.g., removing gas after formation or others). After cycling, coin cells were disassembled for postmortem analysis in a dry argon filled glove box. Anodes and cathodes were washed with dry DMC (three times) and stored inside of the Argon‐filled glovebox.

### Characterization

All cells were tested with a combined performance and durability test. Unless described differently, the voltage window was 3.5–4.9 V. All voltages are referred to the potential difference between the anode and cathode in the electrochemical cell. After two formation cycles at 0.1/0.1 C for charging/discharging (referring to a theoretical capacity of 147 mAh g^−1^), five cycles were run with 0.5/0.5 C. Discharge rates were then increased to 1 C and 2 C for four cycles each. After two more cycles at 0.2/0.2 C, each four cycles with discharge rates of 5 C and 10 C followed. The stability then was tested over 1000 cycles. For charging, a CCCV mode with an initial current rate of 1.5 C followed by a constant voltage step to a current of 0.15 C was applied. A detailed overview of the cell test program is given in the Supporting Information in Table S3 (Supporting Information). Pouch cells were cycled at 23 °C with a BT2000 Battery tester (Arbin Instruments, USA). Coin cells were tested on a CTS Lab Battery tester (BasyTec, Germany) at 26 °C. Full cell tests at elevated and reduced temperatures of 45 and 10 °C were carried out using CR2032 coin cells cycled at MPG‐2 and VMP‐3e potentiostats, respectively (both Biologic, France).

The adhesion strength of cathode layers was determined in a zwikiLine Z2.5/TN with 10N load cell (Zwick‐Roell) via 90° peel tests, based on DIN EN 28510‐1. From electrode stripes with 17 mm width, the aluminum foil was stripped at a constant speed of 600 mm min^−1^. The averaged force between 20 mm and 30 mm distance from the edges was used for strength evaluation.

The ohmic resistivity of the cathodes before and after calendering was measured by placing discs (*∅* = 14 mm) which were cut from electrode sheets between polished copper cylinders (*∅* = 14 mm). A pressure of 6.5 kPa was applied on the cylinders while the through‐plane resistance was measured by a milliohm meter (RM3544, Hioki E.E. Corp., Japan).

The particle morphologies and cathode cross sections were studied by scanning electron microscopy (SEM) with a Zeiss Supra 55 FE‐SEM. The cross‐sections were prepared by an ion‐milling process using argon‐ions (TIC‐3X, Leica Microsystems). For energy‐dispersive X‐ray spectroscopy (EDX) analysis an Ultim Extreme silicon drift detector (Oxford Instruments, UK) with AZtec software was used at an SEM acceleration voltage of 4 kV.

The elements Li, P, Ti, Mn, Fe, and Ni were determined by ICP‐OES with an iCAP 7600 DUO spectrometer (ThermoFisher Scientific, USA). About 35 mg of the samples (weighing accuracy ± 0.04 mg) were extracted in 6 mL hydrochloric acid, 2 mL nitric and 1 mL hydrofluoric acid at 353 K for 4 h in a graphite oven (EasyDigest, Analab). The analysis of the elements was accomplished with four different calibration solutions and an internal standard (Sc). The range of the calibration solutions did not exceed a decade. The two or three major wavelengths of the elements has been used for calculation.

As prepared and postmortem cathodes were investigated by means ofPXRD with a D8‐Advance Bragg‐Brentano diffractometer (Bruker, Germany) using Cu K*α* radiation. Cathode plates with diameters of 16 mm were fixed with adhesive tape on flat specimen holders. The diffraction patterns obtained were refined using the Rietveld method with the Topas 6 Software.

### Statistical Analysis

Data were collected using the measurement equipment described above. The coin cell tests were verified using at least three identically prepared cells. The variation between cells was less than 5%. Characteristic SEM images are shown. These were verified on additional surfaces (at least five different areas per sample). No significant deviations were found on the same sample. The reported Coulomb efficiencies per cathode layer (Table 3) are given for a single measurement. Deviations for identical samples are less than 2% in this case. The pouch cell behavior (Figure 6b) was investigated using one pouch cell each. The ICP measurements (Figure 11) were performed as single determinations on two identical samples, where the elements Li, P, Ti, Mn, Fe, Ni, and Cu were determined. The deviation between the two measurements per sample from the mean value was <10%. OriginPro was used for data analysis.

## Conflict of Interest

As declared in the manuscript, KIT has filed a patent on the additive admixture process for the cathode preparation, which we invented. This simple process can replace complicated coating processes of battery active materials. We first describe and explain this approach in this work. The scientists involved are A.H., P.S., and M.M.

## Author Contributions

A.H.: Resources (equal), Conceptualization (equal), Investigation (equal), Formal Analysis (equal), Writing – Original Draft Preparation (supporting), Writing – Review & Editing (equal), Supervision (equal). J.B.: Resources (equal), Writing – Review & Editing (equal). M.M.: Conceptualization (supporting), Investigation (equal), Formal Analysis (supporting), Writing – Original Draft Preparation (supporting), Writing – Review & Editing (equal). P.S.: Conceptualization (equal), Investigation (equal), Formal Analysis (equal), Writing – Original Draft Preparation (lead), Visualization, Writing – Review & Editing (equal), Supervision (equal). T.B.: Investigation (supporting), Writing – Review & Editing (supporting).

## Supporting information

Supporting Information

## Data Availability

The data that support the findings of this study are available from the corresponding author upon reasonable request.

## References

[1] J. M. Tarascon , E. Wang , F. K. Shokoohi , W. R. McKinnon , S. Colson , J. Electrochem. Soc. 1991, 138, 2859.

[2] K. Amine , H. Tukamoto , H. Yasuda , Y. Fujita , J. Electrochem. Soc. 1996, 143, 1607.

[3] K. Mizushima , P. C. Jones , P. J. Wiseman , J. B. Goodenough , Mater. Res. Bull. 1980, 15, 783.

[4] a) S. Miyai , Positive electrode active material LiCoO_2_ for lithium secondary battery, method for producing the same, and lithium secondary battery , Sony Corp, Japan 1990;

[5] T. Ohzuku , Y. Makimura , Chem. Lett. 2001, 30, 642.

[6] J. Dunn , M. Slattery , A. Kendall , H. Ambrose , S. Shen , Env. Sci. Technol. 2021, 55, 5189. 33764763 10.1021/acs.est.0c07030

[7] P. Stüble , V. Mereacre , H. Geßwein , J. R. Binder , Adv. Energy Mater. 2023, 13, 2203778.

[8] G. Liang , V. K. Peterson , K. W. See , Z. Guo , W. K. Pang , J. Mater. Chem. A 2020, 8, 15373.

[9] W. Li , Y.‐G. Cho , W. Yao , Y. Li , A. Cronk , R. Shimizu , M. A. Schroeder , Y. Fu , F. Zou , V. Battaglia , A. Manthiram , M. Zhang , Y. S. Meng , J. Power Sources 2020, 473, 228579.

[10] H. Xu , H. Zhang , J. Ma , G. Xu , T. Dong , J. Chen , G. Cui , ACS Energy Lett. 2019, 4, 2871.

[11] R. Jung , M. Metzger , F. Maglia , C. Stinner , H. A. Gasteiger , J. Phys. Chem. Lett. 2017, 8, 4820. 28910111 10.1021/acs.jpclett.7b01927

[12] M. Metzger , C. Marino , J. Sicklinger , D. Haering , H. A. Gasteiger , J. Electrochem. Soc. 2015, 162, A1123.

[13] a) C. Arbizzani , F. d. Giorgio , M. Mastragostino , J. Power Sources 2014, 266, 170;

[14] a) P. Stüble , H. Geßwein , S. Indris , M. Müller , J. R. Binder , J. Mater. Chem. A 2022, 10, 9010;

[15] a) J. Chen , Z. Huang , W. Zeng , F. Cao , J. Ma , W. Tian , S. Mu , ChemElectroChem 2021, 8, 608;

[16] E. Hu , S.‐M. Bak , J. Liu , X. Yu , Y. Zhou , S. N. Ehrlich , X.‐Q. Yang , K.‐W. Nam , Chem. Mater. 2014, 26, 1108.

[17] Z. A. Qureshi , H. A. Tariq , R. A. Shakoor , R. Kahraman , S. AlQaradawi , Ceram. Int. 2022, 48, 7374.

[18] a) J. Chong , S. Xun , J. Zhang , X. Song , H. Xie , V. Battaglia , R. Wang , Chemistry 2014, 20, 7479; 24782138 10.1002/chem.201304744

[19] J. Asenbauer , T. Eisenmann , M. Kuenzel , A. Kazzazi , Z. Chen , D. Bresser , Sustainable Energy Fuels 2020, 4, 5387.

[20] U. Farooq , C.‐H. Doh , A. Pervez Syed , D.‐H. Kim , S.‐H. Lee , M. Saleem , S.‐J. Sim , J.‐H. Choi , Carbon Lett. 2016, 17, 39.

[21] N. I. P. Ayu , E. Kartini , L. D. Prayogi , M. Faisal , Supardi , Ionics 2016, 22, 1051.

[22] P. Stüble , J. R. Binder , H. Geßwein , Electrochem. Sci. Adv. 2022, 2, e2100143.

[23] M. Kunduraci , G. G. Amatucci , J. Electrochem. Soc. 2006, A1345.

[24] a) C. Jayawardana , N. Rodrigo , B. Parimalam , B. L. Lucht , ACS Energy Lett. 2021, 6, 3788;

[25] M. Hofmann , F. Nagler , U. Guntow , G. Sextl , G. A. Giffin , J. Electrochem. Soc. 2021, 168, 060511.

[26] Y. Jia , H. Zhong , Z. Wang , Solis State Ionics 2022, 386, 116051.

