# Supplementary material for: Cycling Stability of Lithium‐Ion Batteries Based on Fe–Ti‐Doped LiNi0.5Mn1.5O4 Cathodes, Graphite Anodes, and the Cathode‐Additive Li3PO4
Source: Adv Sci (Weinh). 2023 Jun 22;10(24):2301874. doi: 10.1002/advs.202301874 (PMC10460850; doi:10.1002/advs.202301874)
Supplement: Supplementary file 1 — Supporting Information [file ADVS-10-2301874-s001.pdf]

ADVANCED SCIENCE
Open Access

Supporting Information

for *Adv. Sci.*, DOI 10.1002/advs.202301874

Cycling Stability of Lithium-Ion Batteries Based on Fe–Ti-Doped $LiNi_{0.5}Mn_{1.5}O_4$ Cathodes, Graphite Anodes, and the Cathode-Additive Li_3PO_4

Pirmin Stüble, Marcus Müller, Thomas Bergfeldt, Joachim R. Binder and Andreas Hofmann**

Supporting Information

Cycling stability of lithium-ion batteries based on Fe-Ti doped $LiNi_{0.5}Mn_{1.5}O_4$ cathodes, graphite anodes and the cathode additive Li_3PO_4

Pirmin Stüble[1,2], Marcus Müller[1], Thomas Begfeldt[1], Joachim R. Binder[1], Andreas Hofmann[1]**

Table S1: Detailed information on the auxiliary components of the cathodes and anodes.

Component	Type	Manufacturer
Cathode		
Active Material	LNMFTO	own synthesis
Carbon Black	C-NERGY™ SUPER C65	Timcal/Imerys, France
Graphite	AGB1010	Superior Graphite Co., USA
Binder (PVDF)	Solef 5130	Solvay, Belgium
Anode		
Graphite	SMG-A	Hitachi Chemical, Japan
Carbon Black	C-NERGY™ SUPER C65	Timcal/Imerys, France
Binder A (Na-CMC)	CRT 2000 PA7	DOW Wolff, Germany
Binder B (SBR)	TRD 2001	JSR Micro, Belgium

Table S2: Key results of the cell tests with the corresponding Figure numbers and cycling temperatures. The 1000 cycle capacity retention ("cap. ret.") was calculated from the discharge capacities of the 28th and 1027th cycle. The average capacity retention per cycle ("cap. ret. / cycle")was calculated according to: (cap.ret. 1000 cyc.)$^{0.001}$.

Cathode		Fig.	Temp. [°C]	Cell Type	| -Specific Capacity- | 2nd cycle (0.1C) |-	28th cycle (1.5C) mAh/g	1027th cycle (1.5C) -|	cap. ret. (1000 Cyc.)	cap. ret./ cycle
B	LNMFTO	6	26	CR2032	123.7	120.0	92.3	76.9%	99.974%
C	LNMFTO+1%Li_3PO_4	6	26	CR2032	126.9	120.7	107.1	88.7%	99.988%
D	LNMFTO+2%Li_3PO_4	6	26	CR2032	123.9	119.3	102.5	85.9%	99.985%
G	LNMFTO+1%Li_3PO_4	6,7	26	CR2032	127.3	121.0	109.1	90.2%	99.990%
E	LNMFTO	6	23	Pouchbag	128.8	122.8	97.3	79.2%	99.977%
F	LNMFTO+1%Li_3PO_4	6	23	Pouchbag	128.5	119.7	106.0	88.6%	99.988%
E	LNMFTO	8	26	CR2032	125.7	120.3	92.4	76.8%	99.974%
F	LNMFTO+1%Li_3PO_4	8	26	CR2032	126.9	119.3	105.8	88.6%	99.988%
G	LNMFTO+1%Li_3PO_4	7	10	CR2032	118.5	98.9	96.8	97.8%	99.998%
G	LNMFTO+1%Li_3PO_4	7	45	CR2032	123.7	107.0	49.8	46.6%	99.924%

Table S3: Detailed information on the LNMFTO full cell tests. The (dis)charge modes "CC" and "CCCV" stand for "constant current" and "constant current-constant voltage", respectively. The minimum current for the constant voltage step was 0.05 C. All currents refer to the theoretical capacity of 147 mAh/g. After cell formation and standard cycling (step 1 and 2), a rate capability test was performed (steps 3 to 8). Cycling stability was then tested over 1000 cycles (Step 9). To measure the total capacity with reduced cell polarization effects, test steps with each tow cycles at 0.2/0.2 C were inserted (step 5, 8 and 10). Total test time was roughly 2.5 to 3.5 month, depending on the individual cell performance.

Step	Number of cycles	First cycle #	Last cycle #	Charge mode	Charge rate [C]	Discharge mode	Discharge rate [C]
1	2	1	2	CC	0.1	CC	0.1
2	5	3	7	CC	0.5	CC	0.5
3	4	8	11	CC	0.5	CC	1.0
4	4	12	15	CC	0.5	CC	2
5	2	16	17	CC	0.2	CC	0.2
6	4	18	21	CC	0.5	CC	5
7	4	22	25	CC	0.5	CC	10
8	2	26	27	CC	0.2	CC	0.2
9	1000	28	1027	CCCV	1.0(0.05)	CC	1.5
10	2	1028	1029	CC	0.2	CC	0.2

Table S4: Detailed results from the post mortem ICP-OES analysis: Transition metal content (wt%) of anodes cycled against cathodes E and F after the 1029 cycle test program specified in Table S3.

**Metal**	**Anode 1**	**Anode 2**	**Anode 3**	**Anode 4**
	cycled vs. cathode E (LNMFTO)		cycled vs. cathode F (LNMFTO + 1 % Li_3PO_4)	
Mn	0.0606(12)	0.0593(11)	0.0103(5)	0.0088(4)
Ni	0.0074(4)	0.0074(4)	0.0030(3)	0.0033(2)
Fe	0.0073(7)	0.0159(5)	0.0014(3)	0.0039(4)
Ti	0.0032(2)	0.0030(2)	0.0020(1)	0.0020(1)

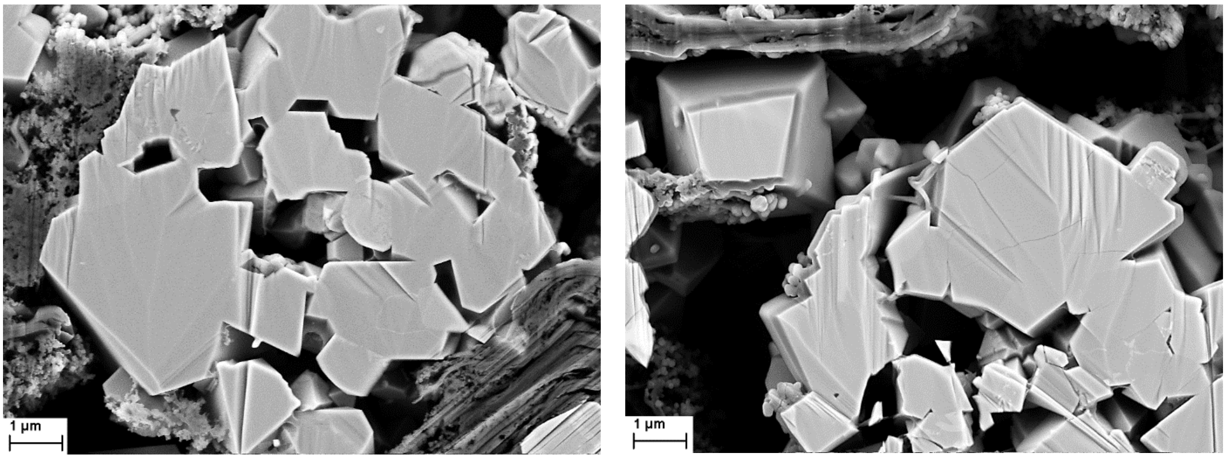

Figure S1: Comparison of the cross sections of LNMFTO granules of cathode B before cycling (left) and after 1029 cycles (right). In the cyceled cathode, noumerous cracks can be observed in the large primary particles.

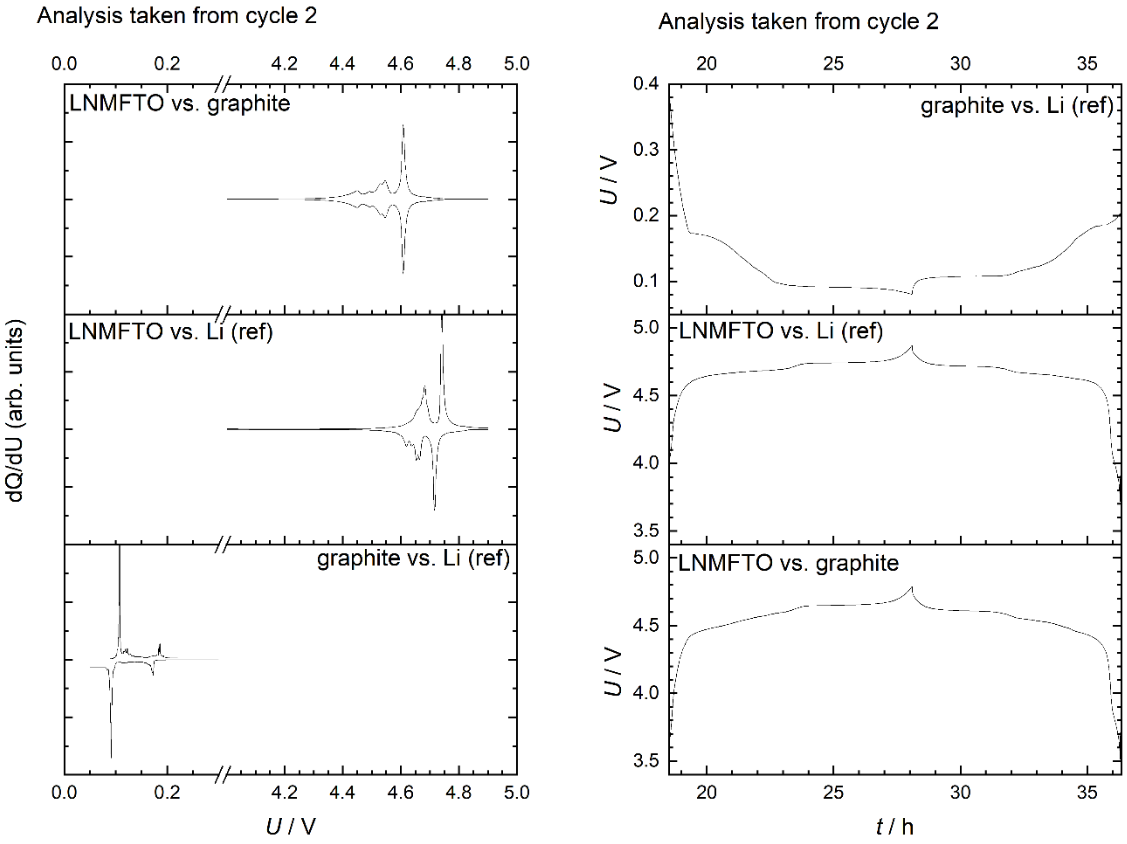

Figure S2: Cell characterisation with three-electrode setup (Cathode B).
